# On the Recycling of Water Atomized Powder and the Effects on Properties of L-PBF Processed 4130 Low-Alloy Steel

**DOI:** 10.3390/ma15010336

**Published:** 2022-01-04

**Authors:** Marawan Abdelwahed, Riccardo Casati, Anna Larsson, Stefano Petrella, Sven Bengtsson, Maurizio Vedani

**Affiliations:** 1 Department of Mechanical Engineering, Politecnico di Milano, 20156 Milan, Italy; riccardo.casati@polimi.it (R.C.); maurizio.vedani@polimi.it (M.V.); 2 Department of Design and Production Engineering, Faculty of Engineering, Ain Shams University, Cairo 11517, Egypt; 3 Höganäs AB, 26383 Höganäs, Sweden; anna.larsson@hoganas.com (A.L.); sven.bengtsson@hoganas.com (S.B.); 4 Certema SCARL, 58044 Cinigiano, Italy; s.petrella@laboratoriotecnologicogrosseto.it

**Keywords:** laser powder bed fusion, water atomized powder, low-alloy steel, powder recycling, carbides

## Abstract

The microstructure and mechanical properties of a 4130-grade steel processed by L-PBF using a feedstock of low-cost water atomized powder have been investigated considering the effects of powder recycling. Chemical analysis of the recycled powder showed a constant amount of alloying elements with a slight reduction in oxygen content. The as-built microstructure was mainly composed of a martensitic structure separated by a high fraction of low-angle grain boundaries, suggesting the application of a direct tempering treatment starting from the as-built condition as a cost-effective post-process thermal treatment rather than the conventional quench and tempering treatment. Moreover, the degree of anisotropy generated by L-PBF in as-built specimens could be reduced after performing either the direct tempering or the quench and tempering treatments. The possible degradation of powder properties on the steel performance was also investigated. After various powder recycling events, no significant deterioration in tensile properties was measured, indicating that the water atomized powder could be a sustainable feedstock candidate for L-PBF.

## 1. Introduction

The laser powder bed fusion (L-PBF) process offers several advantages to the additive manufactured (AM) metallic components, such as the ability to fabricate complex-shape objects with outstanding properties. However, most of the published investigations and known L-PBF applications are confined to aluminum, titanium, and nickel alloys, along with stainless steels and maraging grades [1,2,3]. Low-alloy steels are also potentially attractive due to their low cost and tunable mechanical properties especially after the hardening treatment of quench and tempering (Q & T). Even though low-alloy steels are widely used for structural applications in several industrial sectors, a fairly low number of investigations have been carried out so far on their properties after L-PBF processing [4,5,6,7,8,9,10,11,12,13,14]. The available information showed that the L-PBF processed steels in as-built condition provide outstanding mechanical properties. Dilip et al. [5] as well as Zumofen et al. [10] reported directional dependent tensile properties in as-built condition, suggesting the need of a Q & T thermal treatment to suppress this anisotropy. For those steels exhibiting a martensitic structure right after the L-PBF owing to the rapid cooling conditions, the direct tempering of the as-built microstructure could be proposed as an alternative and cheaper treatment [15].

It is to remark that most of the above-mentioned steels have been processed by L-PBF starting from gas atomized (GA) powders. It is recognized that the water atomization (WA) process could potentially promote cost savings for large batch productions, compared to the standard GA process [16,17]. In a previous investigation [15], it was shown that the L-PBF WA 4130 steel could deliver good hardness and tensile properties, with a slight decrease compared to counter parts fabricated by GA 4130 powder.

The recyclability of powders is also an attractive topic for sustainability issues and environmental considerations. Numerous studies have been performed on the recycling of 316L, 17-4PH, and maraging steel powders for L-PBF [18,19,20]. Although the authors detected both spherical and irregular-shaped spatter particles with oxidized surfaces in the recycled powder batches, the L-PBF processing with re-used powders generally showed overall tensile properties comparable to those of the fresh powder [18,20]; however, Ahmed et al. [19] measured a 7% reduction in ductility after extensive recycling of a 17-PH steel powder. Considering the low-alloy steels, Jelis et al. [21] reported high oxygen levels in recycled 4340 steel powder and measured a significantly lower tensile strength after two re-use cycles. It should be noted that investigations on recycling have been carried out on GA powder so far, while no information is available from the open literature on the behavior of recycled WA powder during L-PBF.

In this context, the current study aims to investigate the microstructure and tensile properties of a 4130 low-alloy steel fabricated by L-PBF, starting from a low-cost WA powder, and evaluating the effects of several recycling events on the achievable steel properties. Another aspect of this investigation is the proposal of a single-step treatment consisting of a direct tempering of the as-built specimens, as a cost-effective alternative to the conventional quench and tempering treatment. Finally, the orientation-related tensile properties have been also considered in the investigation for the above perspectives.

## 2. Materials and Methods

**Figure 1:**
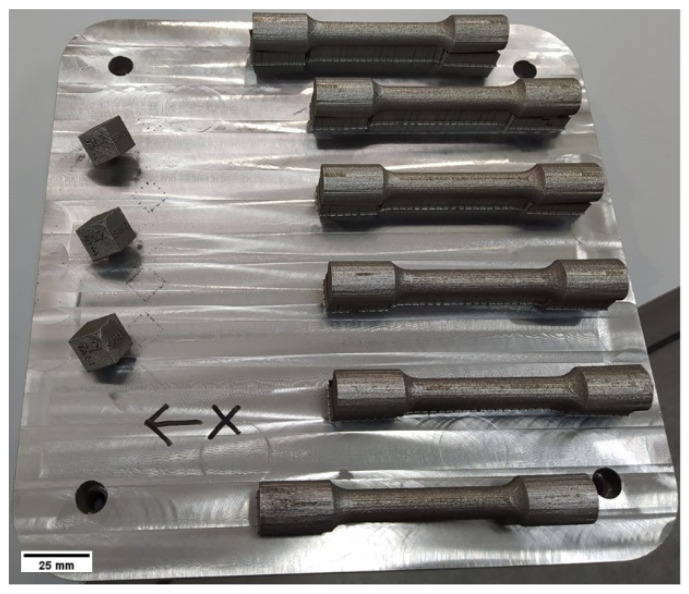
L-PBF build job of W-4130 steel showing the horizontal tensile specimens and the powder capsules.

A feedstock powder of type 4130 low-alloy steel (0.30% C, 1.10% Cr, 0.28% Mo, 0.45% Si, 0.04% Mn, <0.01% S, and 0.29% O) was used for this investigation. The powder was produced by water atomization and subjected to a mechanical post-atomization treatment to improve the morphology of the powder particles. The detailed powder characteristics have been published in a previous research paper [15]. The evolution of chemical composition of the recycled powder was monitored by collecting sealed boxed (cubes with side 15 mm, as shown in Figure 1) of the powder laid on the L-PBF bed. The C, S, O, and N contents were systematically measured by using the Leco model CS-844 as well as model ON-836 for the latter two elements.

A Concept Laser M2 Cusing L-PBF machine was used to fabricate the steel specimens. A nitrogen atmosphere was used in the build chamber, and the optimal processing parameters were defined as 200 W for the laser power, 40 μm for the powder layer thickness, 70 μm and 550 mm/s for the hatching distance and the laser scanning velocity, respectively, according to a previous investigation [15].

The performance of reused WA powder was investigated by designing three recycling events. An industrial approach was followed according to which the remaining powder after each build job (called a “run”) was sieved to cut particles larger than 63 μm and mixed with equal ratio of virgin powder. Therefore, run #1 refers to the L-PBF processing of virgin powder only, while run #4 corresponds to the 3rd recycled set of powder.

The microstructure and chemical composition of both powders and fabricated specimens was characterized by a Zeiss Sigma 500 VP field-emission scanning electron microscope (FE-SEM, Carl Zeiss Microscopy GmbH, Jena, Germany) equipped with energy dispersive spectrometer (EDS). Electron backscattered diffraction (EBSD, Oxford instruments, High Wycombe, United Kingdom) analyses were performed by an accelerating voltage of 20 KV and a 70 nm step size. The measurements were also used to identify and quantify the existing carbides based on their crystal structures. The acquired data have been analyzed by using Channel 5 suite of Oxford HKL Technology. ThermoCalc software was used to compute the fraction of secondary phases existing under equilibrium as a function of temperature, relying on the TCFE9 database.

The mechanical properties of the L-PBF processed steels were evaluated by tensile testing at room temperature according to. ASTM E8M standard. Cylindrical dog-bone specimens (10 mm in diameter and 45 mm in gauge length) referred to each run were fabricated with longitudinal axis parallel to the two main orientations, namely orthogonal (XY-orientation) and parallel (Z-orientation) to the building direction. Different thermal treatments procedures were considered for this investigation. Water quenching was performed after isothermal soaking for 1 h at 840 °C. Tempering was carried out at 550 °C for 1 h. Selected specimens were investigated after Q & T or just after tempering, starting from as-built condition (AB & T).

## 3. Results and Discussion

### 3.1. Powder Degradation

The compositional variations of C, N, S, and O of the steel powders collected during the different runs are given in Table 1. It is observed that the C content of the recycled WA powder remained substantially constant, while a minor reduction in the amount of O content was detected when increasing the number of recycling runs, also considering that the O content in the virgin powder was 0.29%. As for N and S contents, no specific trend was noticed. It is to remark that, according to the EDS analysis, the amount of other alloying elements was substantially constant among the successive runs.

**Figure 2:**
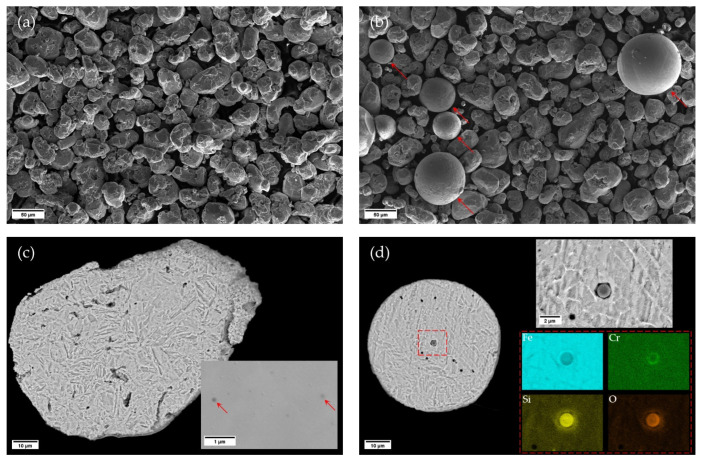
FE-SEM micrographs showing (**a**) virgin powder and (**b**) run #1 powder. Microstructure of (**c**) virgin powder particle and (**d**) spatter particle collected from run #4.

Figure 2a,b display FE-SEM micrographs of the virgin powder and of the powder after run #1, respectively. Few particles with morphology different from the original irregular-shaped WA particles (arrowed in Figure 2b), often with larger size than the average, could be observed. These are believed to be spatters that were generated during the L-PBF process [22]. The recycled powder was sieved before being re-used for the successive L-PBF job to remove the largest spatters. However, particles having diameter smaller than 63 μm could eventually bypass the sieving grid, altering the overall powder characteristics. It is to remark that similar powder morphologies were observed for the subsequent recycling events.

The microstructures of a WA particle and of a spatter particle are displayed in Figure 2c,d, respectively. The high cooling rates produced from the water jets during the atomization process results in a rapid solidification of the virgin powder, which shows a microstructure consisting of a dominant martensitic structure along with some defects as well as nano-size nonmetallic inclusions (marked with arrows in Figure 2c). In spatters a similar microstructure was detected. Moreover, from Figure 2d, few internal porosities were found inside the spatter particle and a micrometer-size oxide inclusion rich in Cr and Si was also observed, as shown in the elemental maps given in the same figure.

### 3.2. Microstructure Evolution

**Figure 3:**
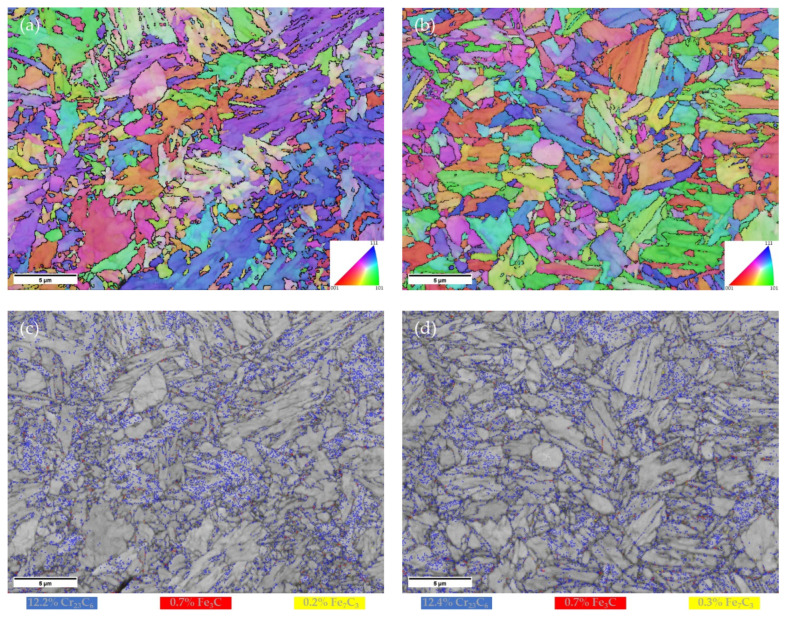
Inverse pole figures (IPF) of the tempered W-4130 steel from (**a**) as-built and (**b**) water-quenched conditions. Band contrast image overlapped by a map of indexed carbides for (**c**) AB & T and (**d**) Q & T steels of run #4.

Figure 3a,b display the microstructure of L-PBF specimens of the investigated steels after tempering, both directly from the as-built state and from the quenched condition, respectively. In both cases, the microstructural features are mainly composed of the martensitic structure. Coarser constituents could be observed in the Q & T state, presumably due to the treatment at 840 °C, while no significant preferential orientation of grains was detected in any condition. In Figure 3c,d, the carbides were indexed based on the identification of their crystal structure. The two tempering regimes result in an almost similar fraction of carbides, which was estimated to be around 13%. However, it is assumed that such evaluation could be overestimated due to carbide size that is smaller than the EBSD pixel size (70 × 70 nm^2^). It is also worth mentioning that the value of 13% corresponds to nearly twice the expected value evaluated by thermodynamic simulations under the hypothesis of equilibrium. Moreover, the equilibrium calculations also expected a mixture of Fe_3_C, M_23_C_6_, and M_7_C_3_ carbides at the same tempering temperature. According to the EBSD analysis, the dominant carbide is of type Cr_23_C_6_ with minor contribution of Fe_3_C and Fe_7_C_3_ that are prone to precipitate around the martensitic boundaries, in both tempers. It is believed that the amount of carbides could vary when considering different positions along the building direction in the AB & T condition owing to different thermal histories experienced during L-PBF [12]. Finally, similar microhardness values of 375 ± 9 HV_0.5_ and 387 ± 4 HV_0.5_ were measured for the AB & T and Q & T steels, respectively.

**Figure 4:**
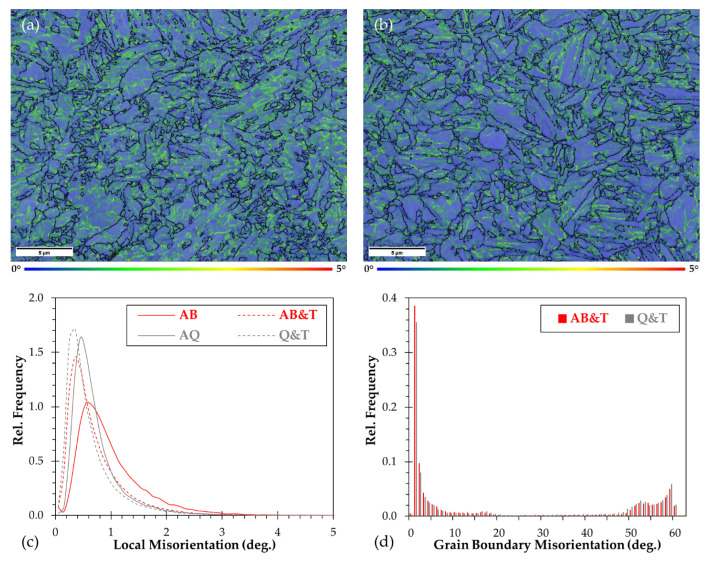
KAM maps of W-4130 steel in (**a**) AB & T and (**b**) Q & T states. Relative frequency of (**c**) local misorientation and (**d**) grain boundary misorientation of the steel in different thermal treatment conditions.

The kernel average misorientation (KAM) maps depicted in Figure 4a,b suggest the local strain distribution within the microstructure after the two treatments investigated. The blue and green colors represent the relatively lower and higher misorientation regions, respectively. An inhomogeneous local strain distribution could be observed within the microstructural constituents that are mainly concentrated between the martensite laths. The quantitative analysis of Figure 4c shows that significantly higher misorientation degrees are produced in the as-built microstructure compared to the as-quenched state, presumably due to the more rapid cooling conditions which arise during the L-PBF rather than the water quenching. As expected, the tempering treatment clearly promotes a reduction in the KAM values with respect to the initial states (both from as-built and from as-quenched states). Further analyses on the grain boundary misorientation are summarized in Figure 4d. The measurements demonstrate that both AB & T and conventionally treated Q & T microstructures are separated by high fraction of low angle grain boundaries (LAGBs < 15°) about 65.3% and 57.6%, respectively. A similar fraction of LAGBs (65%) was measured by Han et al. [12] in as-built 24CrNiMo low-alloy steel, suggesting the finer microstructure generated by the rapid solidification.

### 3.3. Tensile Properties

**Figure 5:**
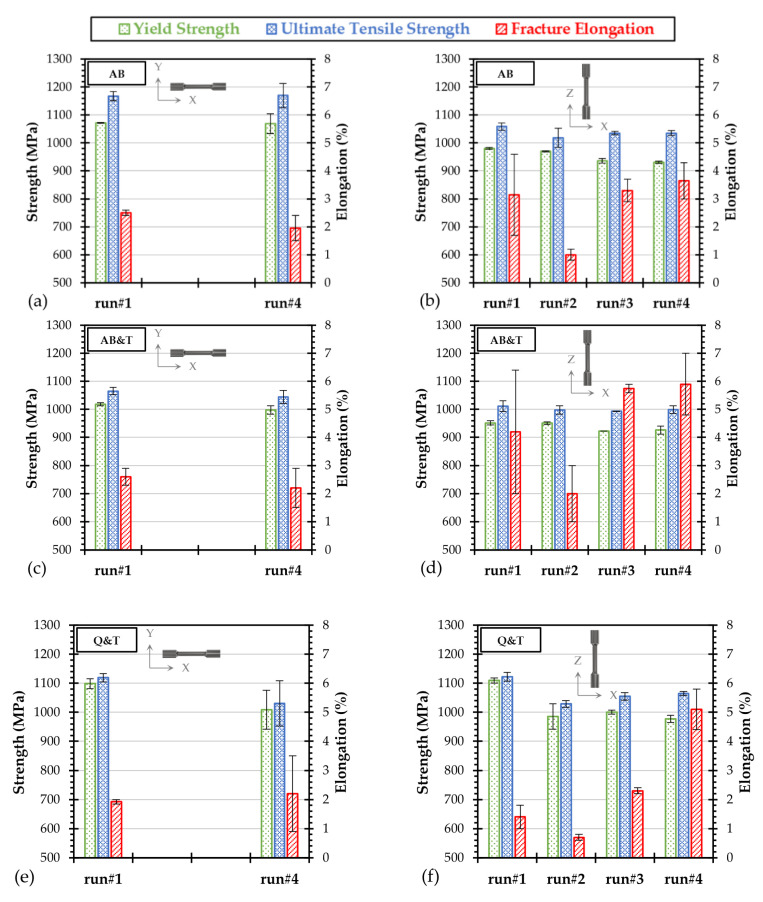
Orientation-related tensile properties of the W-4130 steel processed after powder recycling and tested in (**a**,**b**) AB, (**c**,**d**) AB & T, and (**e**,**f**) Q & T conditions.

The effect of powder recycling on the tensile properties of L-PBF processed WA 4130 steel was investigated considering concurrently the effects of specimen orientation and post treatment conditions on tensile properties. A summary of the tensile data measured on specimens collected from different runs is reported in Figure 5. In any treatment conditions, no significant variations in both yield and ultimate tensile strength could be observed after several recycling events of the WA powder. The tensile strength ranking in XY-direction among the investigated conditions was evaluated from highest to lowest as follows: AB, Q & T, AB & T. When considering the vertical loading direction, the Q & T specimens provided the highest strength followed by the AB and then the AB & T conditions. The lower strength of the AB specimens along the vertical direction is supposed to be due to the intrinsic tempering effect induced upon overlapping of a larger number of layers in the Z-oriented specimens, which results in a more tempered structure compared to the analogous AB XY-specimens [7]. Another possible source of difference comes from the columnar grains that feature a different orientation with respect to the loading-direction [10]. It is to recall that the loss in strength is compensated by an enhanced ductility, which is clearly observed in the behavior of directly tempered specimens.

The average degree of anisotropy in both ultimate tensile strength and fracture elongation has been quantitatively evaluated by considering the ratio between horizontal and vertical properties. A low degree of anisotropy in tensile strength (around 1.12 ± 0.02) was measured for the as-built specimens, since the horizontal specimens always provided higher yield and ultimate tensile strength values, while better ductility (0.66 ± 0.18) was detected for the vertical loading direction. This anisotropy in strength could be reduced to 1.05 ± 0.01 after performing a direct tempering treatment at 550 °C, or even down to 0.98 ± 0.02 after applying the conventional Q & T treatment. However, the direct tempering resulted in an increased ductility anisotropy to reach a value of 0.50 ± 0.17.

## 4. Conclusions

The current study showed the feasibility of recycling a water atomized powder of 4130-alloy steel as a sustainable feedstock for the L-PBF process. The main findings are summarized as follows:

- Spatters generated during the laser processing could be readily found in the feedstock powder right after the first recycling run; however, the pickup of oxygen and the changes in chemistry upon powder recycling was very limited. By increasing the number of recycling events, no substantial variations in the tensile strength could be measured.
- Two different post-process thermal treatments were investigated considering their effects on microstructure and mechanical properties. The as-built microstructure was mainly composed of martensitic laths and blocks separated by a high fraction of low angle grain boundaries (65.3%), while the conventional quench and tempering treatment was able to generate a similar microstructure, yet with a relatively lower fraction (57.6%) of the same grain boundaries.
- Cr_23_C_6_-type carbides were mainly decorating the martensitic lath boundaries with a comparable fraction in both tempered states.
- Anisotropy in mechanical properties was measured for the specimens in as-built condition, which could be reduced by applying a tempering treatment at 550 °C for 1 h. Despite the marginally lower tensile strength shown by the directly tempered specimens, a significant enhancement in ductility was measured compared to the other conditions, offering a promising combination of mechanical properties.

The outcomes of this research highlight that the L-PBF processing of a WA low-alloy steel powder could offer appreciable mechanical properties for structural applications. The ability of the powder to be recycled without showing reduced properties in manufactured parts and the option of using a simpler thermal treatment sequence, based on tempering directly from the as-built state, offer further advantages for the adoption of water atomized low-alloy steel powders within the structural alloys for L-PBF.

## Figures and Tables

**Table 1:** Chemical composition (wt.%) of the W-4130 powder for each recycling set.

	C	N	S	O
Run #1	0.325	0.0055	0.015	0.252
Run #2	0.324	0.0050	0.015	0.254
Run #3	0.324	0.0056	0.015	0.247
Run #4	0.324	0.0054	0.015	0.242

## Data Availability

The raw/processed data required to reproduce these findings cannot be shared at this time as the data also forms part of an ongoing study.
